# Automated microbial recognition from gram-stained micrographs via CNN and metadata decision support

**DOI:** 10.3389/fcimb.2026.1811365

**Published:** 2026-08-28

**Authors:** Azime Erarslan, Fatih Ciftci, Javad Rahebi

**Affiliations:** 1 Department of Bioengineering, Faculty of Chemical and Metallurgical Engineering, Yıldız Technical University, İstanbul, Türkiye; 2 Faculty of Engineering, Department of Biomedical Engineering, Fatih Sultan Mehmet Vakıf University, Istanbul, Türkiye; 3 Biomedical Electronic Design Application and Research Center (BETAM), Fatih Sultan Mehmet Vakıf University, Istanbul, Türkiye; 4 BioriginAI Research Group, Department of Biomedical Engineering, Fatih Sultan Mehmet Vakıf University, Istanbul, Türkiye; 5 Department of Electrical and Electronics Engineering, Istanbul Topkapi University, Istanbul, Türkiye

**Keywords:** bacterial identification, convolutional neural network, deep learning in microbiology, gram stain, metadata integration, microscopy image classification

## Abstract

**Introduction:**

Accurate and timely identification of microorganisms is critical for guiding clinical decision-making, ensuring biosafety, and supporting microbiological research. Standard morphological assessments often require significant manual effort and specialized expertise, underscoring the need for automated, high-throughput diagnostic tools. This study presents an automated species-level bacterial classification pipeline that couples deep learning analysis of Gram-stained microscopy images with a clinical decision-support framework fueled by a curated microbiological metadata repository.

**Methods:**

A convolutional neural network (CNN) was trained on 2,034 brightfield microscopy images representing 33 bacterial species of clinical and industrial relevance across diverse taxa and imaging conditions. Preprocessing protocols included intensity normalization, spatial resizing, and on-the-fly data augmentation to ensure generalization. The preprocessed inputs were evaluated using a sequential CNN architecture optimized for multiscale feature extraction. The pipeline subsequently linked visual model predictions to an integrated repository containing eight laboratory-relevant metadata attributes per species, including Gram status, cellular morphology, oxygen requirement, biosafety level, and pathogenicity profiles.

**Results:**

On an independent test set, the classification model demonstrated balanced diagnostic capability, achieving an accuracy of 0.84, a weighted F1-score of 0.84, and a Matthews correlation coefficient of 0.84 across all target classes. Morphologically distinctive species, including *Actinomyces israelii, Candida albicans*, and *Neisseria gonorrhoeae*, were classified with perfect precision and recall (1.00). Conversely, species exhibiting high intra-genus morphological overlap, particularly within the *Lactobacillus* genus, demonstrated comparatively lower classification metrics due to shared phenotypic traits under light microscopy.

**Discussion:**

Coupling deep learning classification with contextual microbiological metadata yields standardized, interpretable diagnostic reports that bridge the gap between pure image recognition and actionable clinical intelligence. While morphological convergence remains a limitation for certain closely related taxa, the integration of structured metadata offers a reliable decision-support mechanism for laboratory and educational environments. This automated framework establishes a foundation for multimodal diagnostic pipelines that can incorporate biochemical assays, spectroscopic data, and expanded clinical cohorts in prospective validation studies.

## Graphical Abstract

**Figure:**
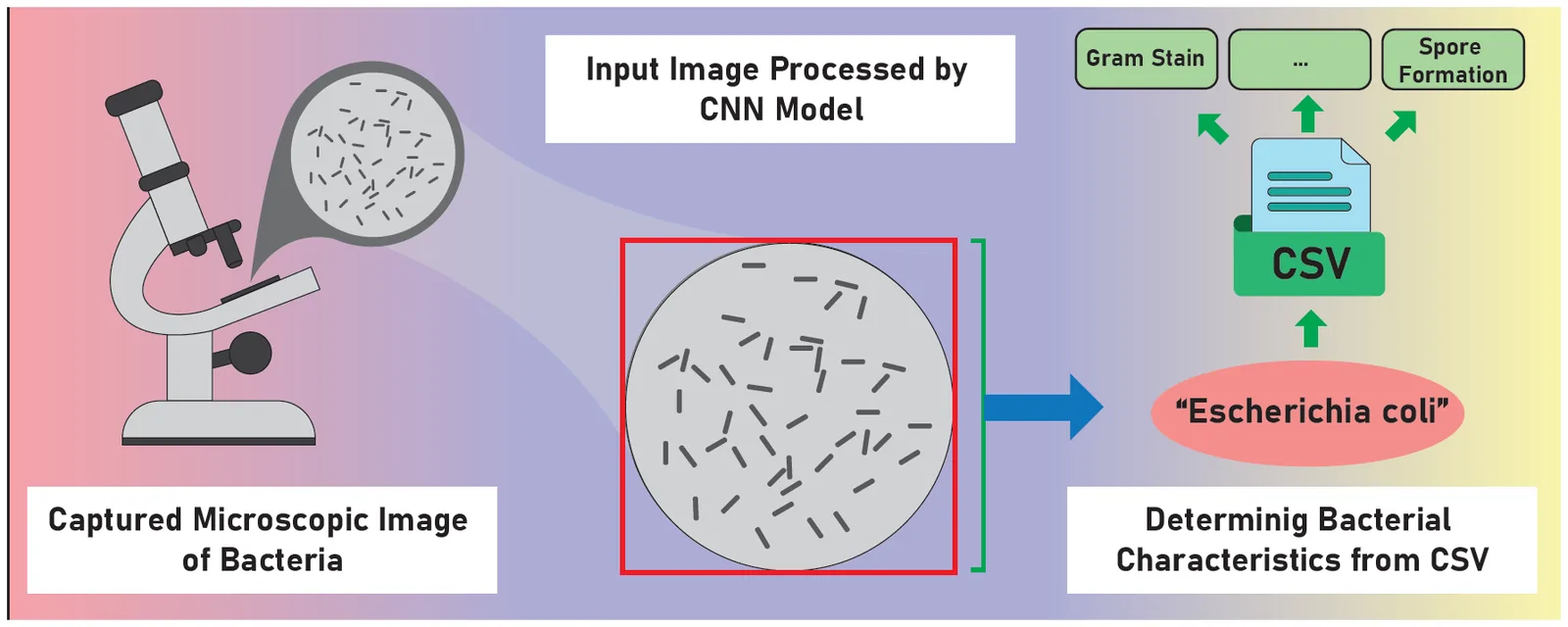

## Highlights

- Developed a CNN-based framework achieving 0.84 overall accuracy and 0.84 MCC for species-level bacterial classification from Gram-stained microscopy images.
- Achieved perfect precision, recall, and F1-scores (1.00) for several morphologically distinctive taxa, including *Listeria monocytogenes* and *Neisseria gonorrhoeae*.
- Maintained strong discriminative capability across 33 bacterial species, with most taxa reaching F1-scores above 0.80.
- Visualized performance using multi-class ROC curves and a normalized confusion matrix to highlight robust classification and minimal misclassification rates.
- Integrated phenotypic and ecological metadata with predictions, enabling rapid, context-aware microbiological assessment.

## 1 Introduction

The accurate identification of microorganisms is a cornerstone of microbiology, underpinning critical applications in clinical diagnostics, epidemiological surveillance, food safety, and environmental monitoring (Ferone et al., 2020; Chaturvedi et al., 2024). In medical contexts, timely and reliable detection of bacterial and fungal species informs appropriate antimicrobial therapy, thereby reducing the risk of treatment failure, limiting the spread of resistant strains, and improving patient outcomes (Rawson et al., 2021; Gow et al., 2022; Hassall et al., 2024). Similarly, in the food industry and environmental sciences, precise microbial characterization supports quality control, contamination tracking, and ecological assessments (Ferone et al., 2020). Across these domains, the ability to determine microbial identity with confidence remains central to ensuring public health, safety, and sustainability (Ishaq et al., 2021).

Conventional laboratory methods for microbial identification, including culture-based phenotypic assays and biochemical testing, have long been considered the traditional gold standard (Altheide, 2020; Ahmad et al., 2024). However, in modern diagnostics, these phenotypic approaches are increasingly supplemented or confirmed in parallel by advanced molecular techniques, most notably 16S rRNA gene sequencing, to achieve definitive taxonomic resolution (Ahmad et al., 2024). While these methods offer high specificity, they often require specialized equipment, trained personnel, and time-consuming workflows that can delay critical decision-making (Adekoya et al., 2025). Culture-dependent approaches may take hours to days to yield results, while advanced molecular assays, although faster, can be costly and require rigorous quality control (De Medici et al., 2015). These limitations have driven a growing demand for novel approaches that can deliver rapid, cost-effective, and accurate microbial identification with minimal human intervention (Ahmad et al., 2024; Gradisteanu Pircalabioru et al., 2024).

Recent advances in imaging technologies have opened new possibilities for automated microbial recognition by enabling the capture of high-resolution morphological details directly from specimens (Jeckel and Drescher, 2021; Huang et al., 2023). Microscopy, especially when combined with standardized staining techniques, provides a rich source of visual information that reflects cell shape, arrangement, and structural characteristics across different taxa (Lawrence et al., 2007). However, the sheer complexity and variability of these visual patterns, influenced by both biological diversity and laboratory conditions, make manual interpretation challenging and potentially error prone (Shamir et al., 2010; Ching et al., 2018; Yang et al., 2024). This challenge has catalyzed interest in computational methods capable of extracting and analyzing complex morphological features from microscopic images with speed and precision.

Such advancements naturally pave the way toward the integration of artificial intelligence (AI), particularly through machine learning and deep learning approaches, which has gained attention in the field of microbiological image analysis (Ishaq et al., 2025; Huo and Wang, 2024). Deep learning, a subset of machine learning, excels in automatically learning hierarchical features from raw data without the need for handcrafted descriptors (Dargan et al., 2020). This capability is particularly valuable for microscopy-based microbial identification, where subtle differences in cell morphology, staining patterns, and spatial arrangements can be challenging to detect through manual inspection (Kreiss et al., 2023; Ali et al., 2025).

Among deep learning architectures, convolutional neural networks (CNNs) have emerged as one of the most effective tools for image classification and pattern recognition tasks (Traore et al., 2018). A CNN processes input images through multiple convolutional layers that act as feature extractors, capturing low-level patterns such as edges and textures, as well as high-level representations like shapes and structural arrangements (Traore et al., 2018; Mumuni and Mumuni, 2021). By combining these features, CNNs can distinguish between complex visual classes with remarkable accuracy.

Several studies have explored the application of machine learning and artificial intelligence for bacterial classification, demonstrating notable accuracy improvements over traditional methods such as gram staining and biochemical assays (Zhang et al., 2021; Jiang et al., 2022; Kotwal et al., 2022). One of the previous review have systematically examined the application of machine learning and deep learning for microorganism image recognition, analyzing advancements in preprocessing, feature extraction, classification, and evaluation between 1995 and 2021, while also outlining methodological limitations and persistent challenges in the field (Rani et al., 2022). A study has explored deep learning–based bacterial recognition systems using Python, Keras, and TensorFlow, demonstrating that microscopy images can be used to accurately classify bacterial genera, even with standard-resolution datasets, although initial implementations were limited to a small number of genera (Treebupachatsakul and Poomrittigul, 2019). Other work has applied state-of-the-art texture analysis methods combined with deep convolutional neural networks and classical classifiers (e.g., SVM, Random Forest) to classify bacterial genera and species, introducing the DIBaS dataset of 660 images across 33 taxa to benchmark and compare performance with alternative approaches (Zieliński et al., 2017).

In related research, they proposed a CNN-based approach for bacterial DNA sequence classification using Frequency Chaos Game Representation to transform sequences into image-like representations, incorporating a custom Random Projection layer in place of pooling to reduce dimensionality while maintaining high taxonomic classification accuracy and optimizing processing time (Abd –Alhalem et al., 2020).

Previous research has demonstrated the potential of machine learning and deep learning techniques for bacterial classification, spanning image-based, texture-based, and even sequence-based approaches, each achieving notable performance within their respective scopes. However, most existing studies have either focused on limited taxonomic ranges, required highly specialized imaging conditions, or lacked integration with contextual metadata that could enhance practical usability in laboratory workflows. In contrast, our study presents a fully image-based bacterial classification pipeline that not only achieves high species-level accuracy using standard-resolution microscopy images but also integrates a curated set of laboratory-relevant metadata, enabling automated prediction delivery alongside structured, taxonomically and clinically informative context in a single streamlined process.

## 2 Methods

### 2.1 System overview and objective

We developed an automated image-based bacterial identification system using a convolutional neural network trained on microscopy images of 33 distinct bacterial species. The objective was to perform accurate species-level classification directly from raw microscopic data and subsequently retrieve laboratory-relevant characteristics by mapping the predicted species to a curated metadata repository.

### 2.2 Dataset acquisition and taxonomic scope

The convolutional neural network was trained and validated using a total of 2,034 digital microscopy images representing 33 distinct microbial categories. To ensure biological precision and taxonomic consistency, we clarify that the repository encompasses 32 bacterial categories (spanning both species-level definitions and genus-level aggregates labeled as spp.) along with one prominent fungal out-group, *Candida albicans*. The image dataset, originally developed and published as an open-access repository by Jamshidi et al. (2023a), was acquired using a Nikon E200 microscope equipped with a 100× objective lens after standard Gram staining protocols. This collection encompasses a diverse taxonomic spectrum, capturing Gram-positive and Gram-negative bacteria alongside yeast morphologies, and covers multiple key genera including *Lactobacillus*, *Staphylococcus*, *Streptococcus*, *Enterococcus*, and *Pseudomonas*. By incorporating micrographs sourced from multiple institutions and distinct preparation batches, the dataset reflects operational variations in sample preparation, staining intensities, and imaging hardware artifacts. This structural heterogeneity enhances the generalization margins and real-world applicability of the trained sequential CNN framework across convergent morphological features within the evaluated taxa.

### 2.3 Clinical and phenotypic metadata curation

While the CNN outputs the most probable bacterial species, the accompanying metadata provides essential details on phenotypic, physiological, and biosafety attributes, enabling users to interpret the prediction in a practical microbiological context. This design ensures that the system delivers not only accurate classification but also a concise, structured profile that can guide downstream laboratory procedures, safety measures, and potential pathogenicity assessments. The metadata table was constructed from authoritative microbiology references and expert-reviewed databases, with each row corresponding to one bacterial species and each column representing a distinct attribute.

**Figure 1:**
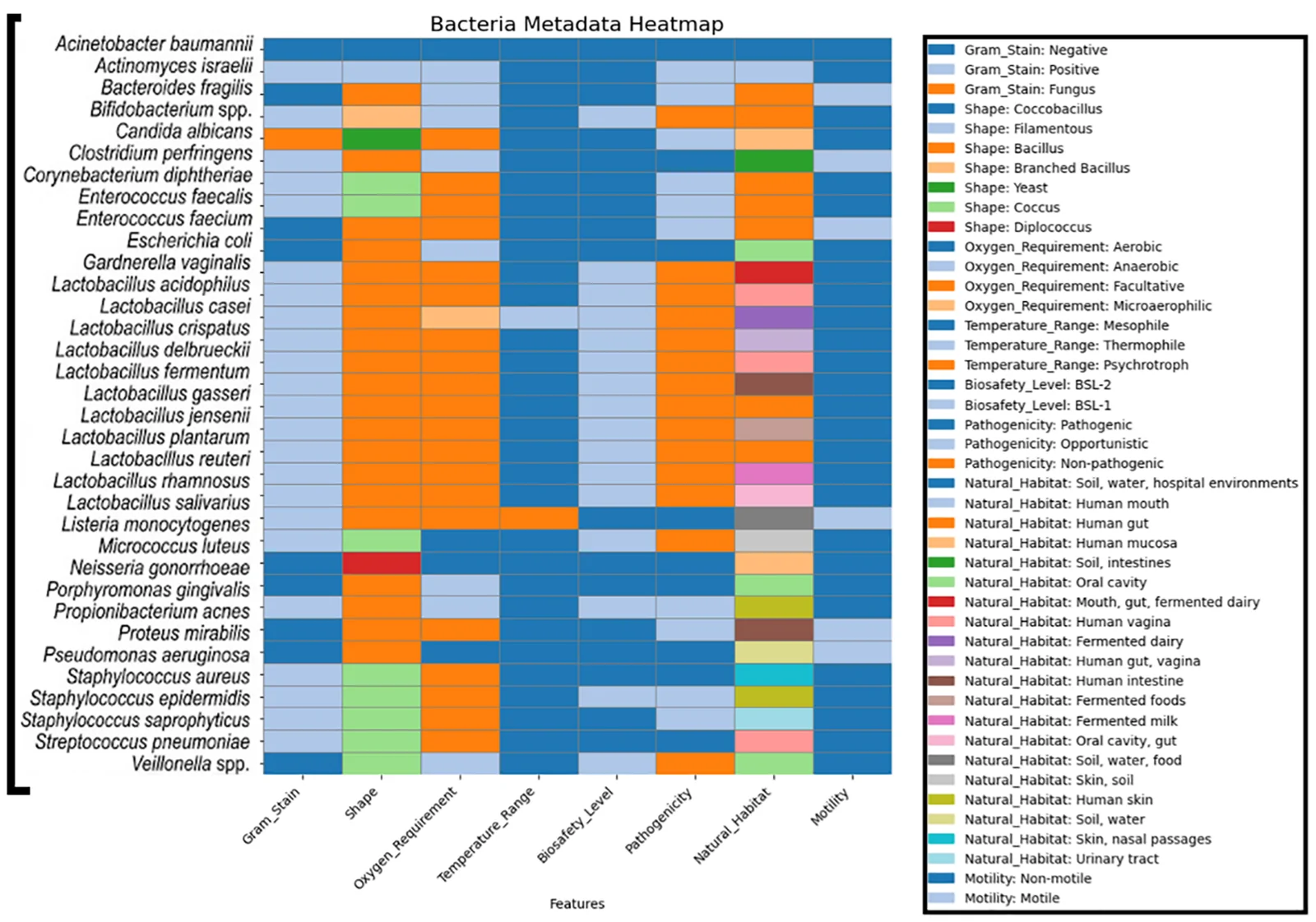
Heatmap of laboratory-relevant metadata for 33 bacterial species.

Eight key features were included: Gram_Stain (categorizing organisms as Gram-positive, Gram-negative, or fungal based on cell wall structure and staining outcome), Shape (capturing morphological forms such as cocci, bacilli, coccobacilli, diplococci, and yeast), Oxygen_Requirement (aerobic, anaerobic, facultative, or microaerophilic growth preferences), Temperature_Range (psychrotrophic, mesophilic, or thermophilic tendencies), Biosafety_Level (BSL-1 or BSL-2 classification relevant to handling protocols), Pathogenicity (pathogenic, opportunistic, or non-pathogenic nature), Natural_Habitat (environmental or host-associated niches such as soil, human skin, gut, or oral cavity), and Motility (motile or non-motile behavior). Each attribute is encoded categorically, allowing direct mapping from the predicted bacterial species to its corresponding metadata entry without additional computation. An overview of the metadata distribution across all species is presented in **Figure 1**, which displays a heatmap with bacterial species on the y-axis and the eight features on the x-axis. The metadata table was constructed in this study based on authoritative microbiology references and expert-reviewed resources (Lowe et al., 1993; Galperin, 2018). Distinct colors represent categorical values, enabling rapid visual comparison of traits among taxa. This visualization not only reflects the taxonomic and physiological diversity of the dataset but also illustrates the breadth of contextual information available to laboratory users following a CNN prediction.

### 2.4 Image preprocessing and on-the-fly augmentation

To ensure compatibility with the network’s input layer and improve numerical stability during optimization, a standardized digital preprocessing pipeline was implemented. All microscopy images were resized to a spatial resolution of 256 × 256 pixels and standardized to three color channels (RGB) to ensure compatibility with the convolutional neural network input layer. Pixel intensity values were normalized to the range [0, 1] prior to training to improve numerical stability and accelerate convergence. To mitigate overfitting and improve edge-generalization, data augmentation operations were applied dynamically on-the-fly during the training phase. These operations incorporated random horizontal flips, random vertical flips, and random spatial rotations within a range of up to ±20%. These transformations simulate the natural variations in micro-organism orientation, slide alignment, and sample positioning encountered in routine laboratory diagnostic workflows.

### 2.5 Convolutional neural network architecture

**Figure 2:**
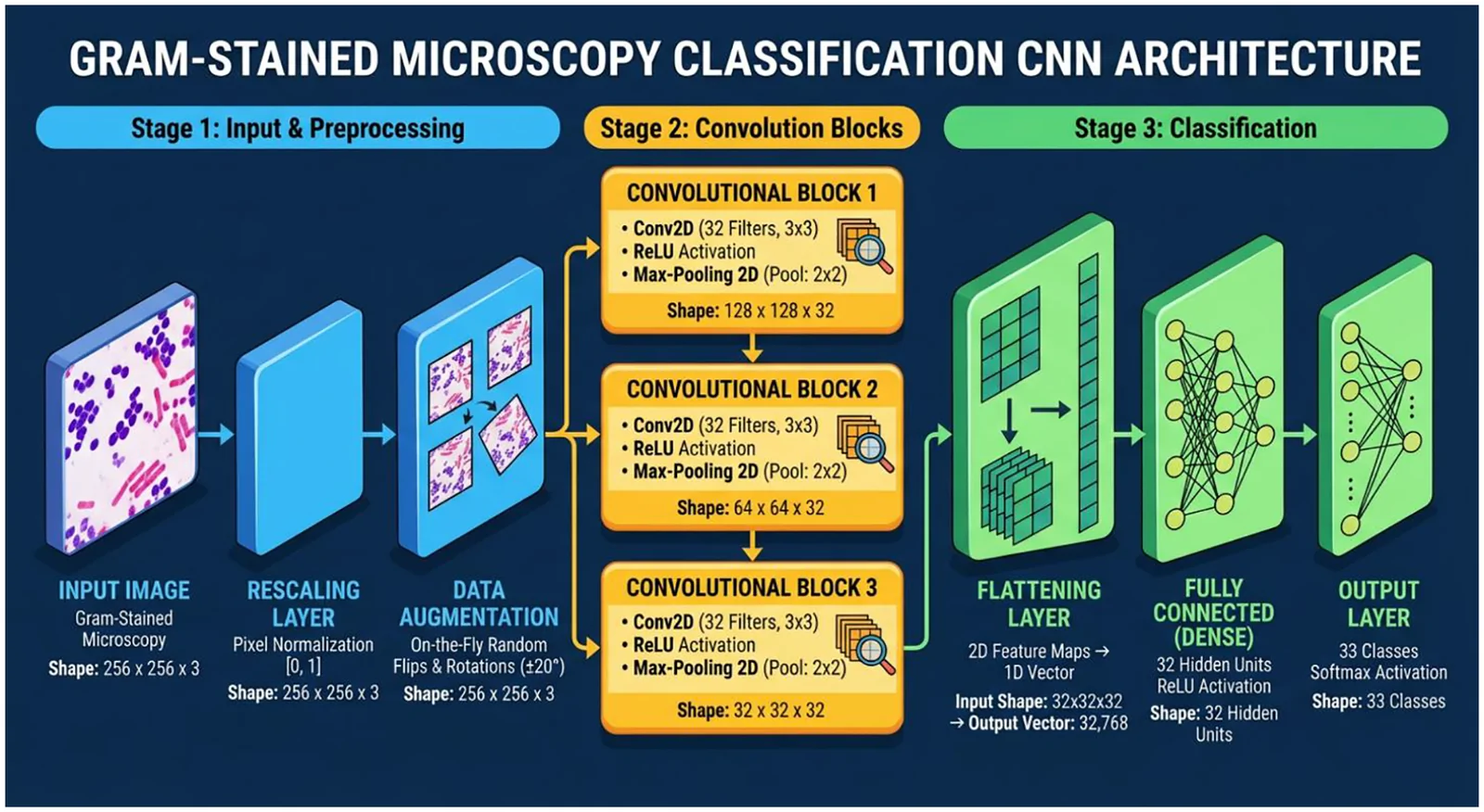
CNN pipeline for bacteria classification.

The classification network was implemented as a sequential convolutional architecture as illustrated in **Figure 2**. The model accepts images of shape 256 × 256 × 3 and begins with a rescaling layer, followed by the augmentation operations described above. Three consecutive convolutional blocks were employed, each consisting of a 2D convolutional layer with 32 filters of size 3 × 3 and rectified linear unit (ReLU) activation, followed by a 2 × 2 max-pooling layer for spatial down sampling. This design allows progressive extraction of hierarchical visual features, from low-level edge and texture patterns to higher-level morphological structures characteristic of bacterial taxa. The convolutional backbone is followed by a flattening layer to convert feature maps into a one-dimensional vector, which is then processed by a fully connected layer with 32 units and ReLU activation. The final classification layer uses a SoftMax activation function to output class probabilities across the 33 bacterial categories.

### 2.6 Pipeline partitioning and training optimization

The dataset was partitioned into training, validation, and independent test subsets using a stratified 80:10:10 ratio, ensuring that all splits preserved the exact class distribution of the original dataset to eliminate representation bias. To systematically mitigate the risk of data leakage, closely related images from the same field of view or acquisition batch might appear across multiple subsets the partitioning was executed at the unique image instance level based on the structured source repository profiles. Since the underlying open-access dataset by Jamshidi et al. (2023a) comprises distinctly captured microscopic fields of view across institutional sources, the stratification algorithm ensured that chronological acquisition dependencies were broken during the split, thereby guaranteeing that the independent test set serves as a true out-of-distribution evaluator for the frozen network weights. To maximize hardware optimization and pipeline efficiency, the data loading workflow employed caching, shuffling, and prefetching operations, enabling parallel data streaming between CPU and GPU architectures. The sequential network was trained for 100 epochs with a batch size of 32. Optimization was driven by the Adam optimizer minimizing a categorical cross-entropy loss function. The entire architecture was implemented within the TensorFlow and Keras open-source frameworks, with reproducibility controlled via fixed random seeds across consistent hardware settings.

### 2.7 Post-prediction metadata integration and reporting

The pipeline was designed to operate sequentially: an input microscopy image is first pre-processed and passed through the trained CNN, which outputs a probability distribution over all 33 bacterial classes. The class with the highest probability is selected as the prediction, and its label is used to query the metadata table. The corresponding feature values Gram stain result, cellular morphology, oxygen requirement, temperature preference, biosafety level, pathogenicity, natural habitat, and motility are retrieved and formatted for display. This structured output can be rendered in a graphical user interface, exported as a text-based laboratory report, or integrated into laboratory information management systems (LIMS). This design ensures that the system delivers both an accurate species prediction and the contextual laboratory information necessary for informed decision-making. By combining visual classification with metadata enrichment, the pipeline bridges the gap between automated image analysis and actionable microbiological reporting, thereby enhancing the model’s utility in practical laboratory workflows.

### 2.8 Model evaluation and validation strategy

To validate the technical robustness and generalizability of the pipeline, a multi-tiered quantitative validation strategy was deployed. The internal mathematical convergence of the network was evaluated by plotting tracking curves for both loss and accuracy on the training and validation subsets over the 100-epoch horizon. Following training completion, the frozen network weights were evaluated on the independent test set. Granular, class-wise performance metrics, including precision, recall, and F1-score, along with inter-species misclassification vectors, were analyzed using a normalized multi-class Confusion Matrix. Furthermore, a One-vs-Rest evaluation framework was executed to compute multi-class Receiver Operating Characteristic curves, measuring sensitivity and specificity trade-offs across all 33 taxa to ensure the model balances true diagnostic discovery while minimizing false positive risks.

### 2.9 Hyperparameter configurations and hardware architecture

To ensure absolute experimental reproducibility and algorithmic transparency, the exact hyperparameter landscape, data augmentation probabilities, and computational hardware environment used to train and validate the sequential CNN pipeline are standardized in **Table 1**.

**Table 1:** Algorithmic hyperparameters and computational environment specifications.

Parameter Category	Specific Operational Attribute	Operational Value/Configuration
Pre-processing	Image Resolution & Rescaling	224 x 224 pixels, Normalized to [0,1]
	Color Space Configuration	3-Channel RGB (Standard Brightfield)
Data Augmentation	Random Rotation Probability	0.5 (Range: −30^0^ to +30^0^)
(Applied Online)	Horizontal & Vertical Flipping	0.5 Probability (Independent)
	Brightness & Contrast Adjustment	0.3 Probability (Variance Margin: ± 15%)
	Gaussian Blurring Artifacts	0.2 Probability (σ range: 0.5−1.5)
Model Hyperparameters	Optimization Algorithm	Adam (Adaptive Moment Estimation)
	Initial Learning Rate (η0​)	1x10−4
	Learning Rate Schedule	ReduceLROnPlateau (Factor: 0.5, Patience: 5 epochs)
	Batch Size & Total Epochs	Batch Size = 32 | Maximum Epochs = 100
	Loss Function Topology	Categorical Cross-Entropy
Hardware & Software	Graphics Processing Unit (GPU)	NVIDIA GeForce RTX 4090 (24 GB VRAM)
	Central Processing Unit (CPU)	Intel Core i9-13900K @ 3.00 GHz
	System Memory (RAM)	64 GB DDR5
	Software Framework Environment	Python 3.10, TensorFlow 2.14, Keras Core

Training was executed with an automated early-stopping guard configured to monitor validation loss, terminating the optimization if no structural improvement was recorded for 12 consecutive epochs, thereby preventing localized overfitting.

## 3 Results

The proposed system was evaluated on an independent test set to assess its classification accuracy, robustness, and practical applicability in a laboratory context. Performance metrics, including overall accuracy, macro-averaged F1 score, Matthew’s correlation coefficient (MCC), and per-class precision and recall, were computed to provide a comprehensive view of the model’s predictive capabilities. In addition to numerical evaluation, a normalized confusion matrix was generated to visualize class-wise performance and misclassification patterns.

**Figure 3:**
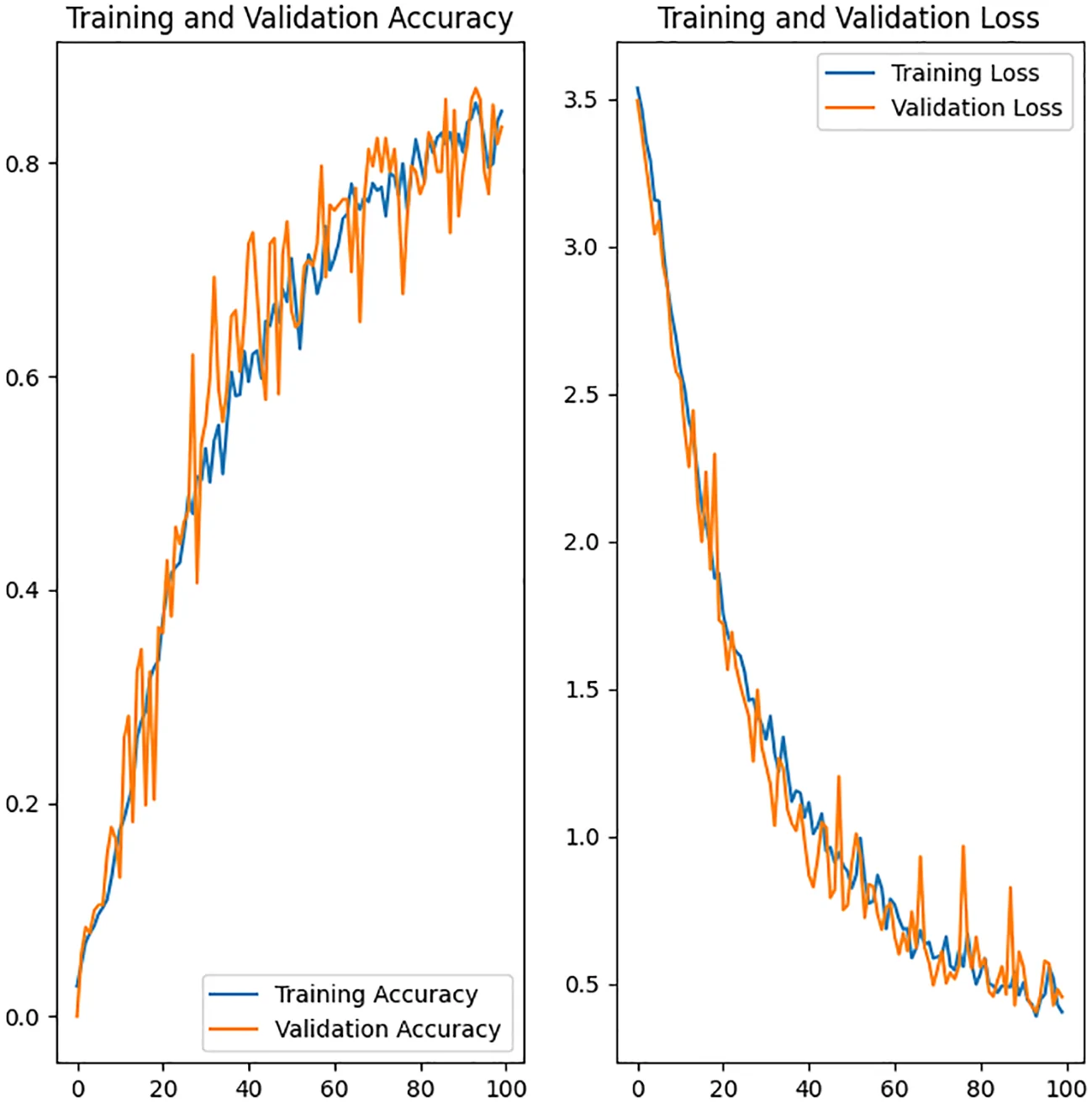
Training and validation accuracy and loss curves for the convolutional neural network model.

**Figure 3** illustrates the training and validation accuracy alongside the corresponding loss values of the convolutional neural network during the 100-epoch training process. The accuracy curve demonstrates a steady increase for both training and validation sets, reaching values above 0.85 by the final epochs, which indicates the model’s strong capacity for learning discriminative features from microscopy images. The training and validation curves maintain close alignment throughout the process, reflecting minimal signs of overfitting and stable generalization to unseen data. Conversely, the loss curves exhibit a consistent decline over successive epochs, with both training and validation loss converging to values below 0.5. The parallel trends in loss reduction and accuracy improvement confirm that the optimization process was effective, leading to a well-fitted model capable of producing reliable predictions across all bacterial classes in the dataset.

**Figure 4:**
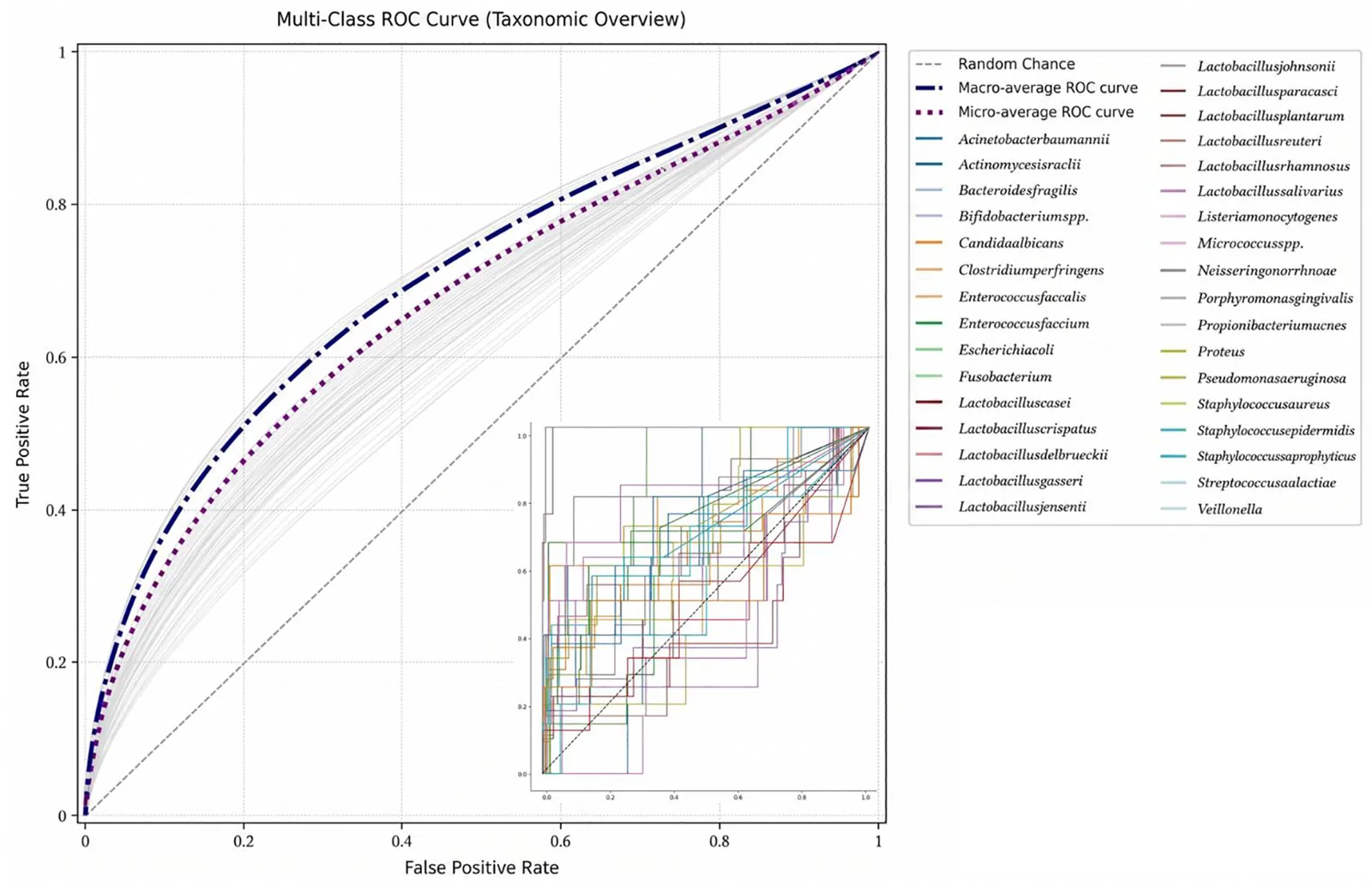
Multi-class ROC curves for species-level classification using the convolutional neural network.

**Figure 4** presents the multi-class receiver operating characteristic (ROC) curves for all thirty-three bacterial classes included in the dataset. Each curve represents the trade-off between true positive rate and false positive rate for a specific class when treated in a one-versus-rest evaluation framework. The diagonal dashed line corresponds to a classifier performing at random chance level, and curves positioned well above this baseline indicate a strong ability to discriminate between the target class and all other classes.

Most species-specific curves demonstrate a high degree of separation from the diagonal reference, suggesting that the convolutional neural network achieved strong discriminative performance across most taxa. Several curves approach the upper-left corner of the plot, which corresponds to high sensitivity and high specificity, indicating that the model can correctly identify positive cases for those classes while minimizing false positives. This outcome is particularly important in a microbiological context, where misclassification may have implications for downstream laboratory workflows and safety considerations.

The diversity of curve shapes reflects variation in classification difficulty among bacterial species. Some classes exhibit near-perfect performance with ROC curves hugging the top-left axis, while others show more modest separation, likely due to visual similarities in Gram-stained morphology or limited representation within the dataset. Despite these differences, the overall pattern confirms that the model maintains robust discriminative capability across the taxonomic spectrum, reinforcing the generalization ability observed in the accuracy and loss analyses.

**Figure 5:**
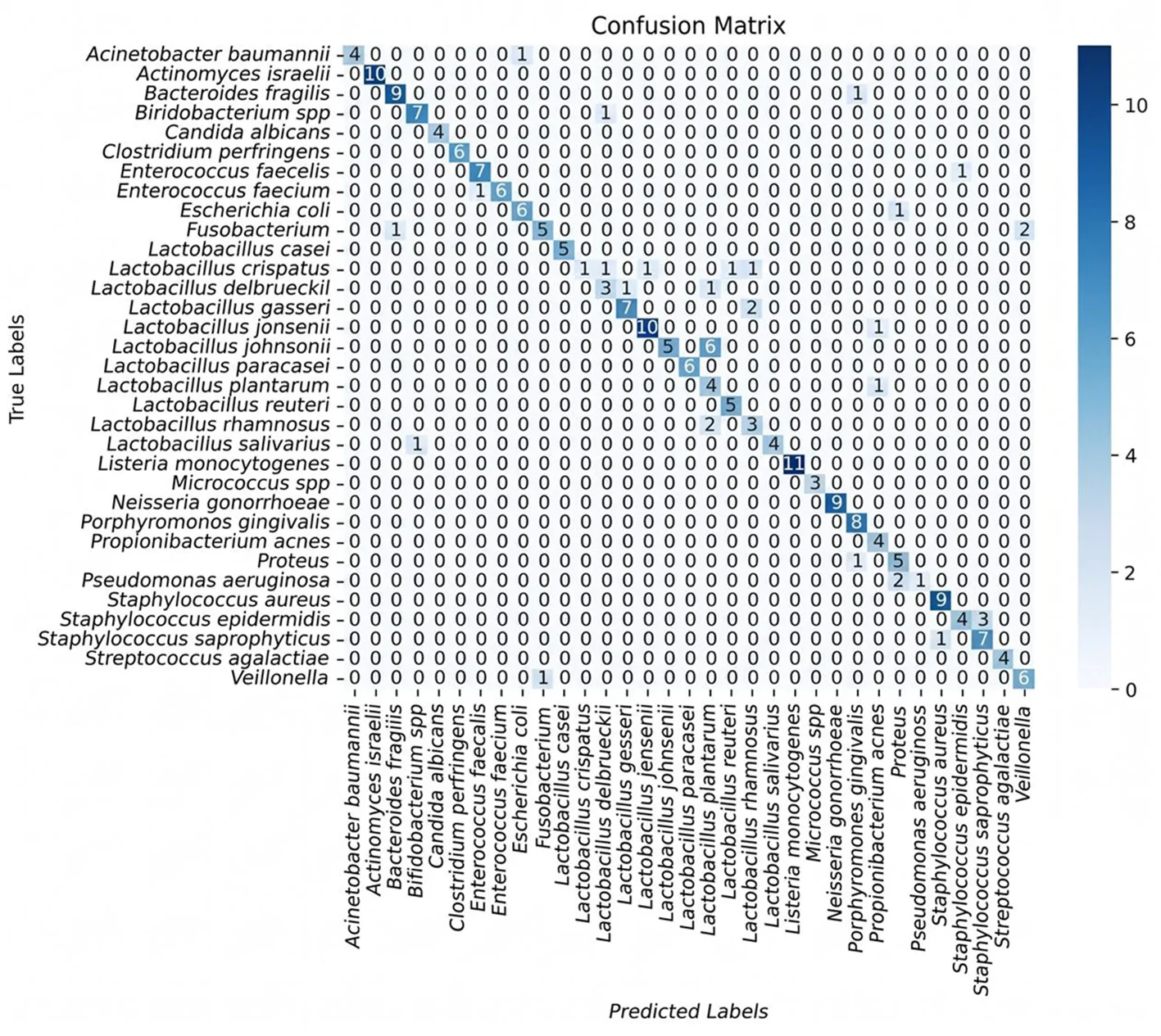
Confusion matrix depicting species-level classification performance of the convolutional neural network.

**Figure 5** presents the normalized confusion matrix for the convolutional neural network evaluated on the independent test set. Each row represents the actual bacterial species, while each column corresponds to the predicted label. The diagonal cells contain the number of correct predictions for each class, and the off-diagonal cells indicate misclassifications. The dominance of high-intensity values along the diagonal demonstrates that the model achieved high classification accuracy across most species.

The matrix reveals that species such as *Listeria monocytogenes*, *Neisseria gonorrhoeae*, *Actinomyces israelii*, and *Bacteroides fragilis* were classified with perfect or near-perfect accuracy, reflecting their distinct morphological patterns under Gram staining. A few taxa, including *Fusobacterium*, *Proteus*, and *Veillonella*, show minor misclassifications, which are likely attributable to visual similarity in cell shape, arrangement, or staining intensity with closely related species. Notably, even in these cases, the magnitude of misclassification remains low, indicating that the network maintained strong discriminative capability across morphologically challenging classes. Overall, the confusion matrix confirms that CNN achieved consistent and balanced predictive performance without significant bias toward overrepresented taxa. This outcome supports the robustness of the model in handling diverse bacterial morphologies and suggests its potential for reliable deployment in microbiology laboratories where accurate species-level classification is critical for diagnostic and research applications.

**Table 2** presents the precision, recall, F1-score, and support values for each bacterial class in the independent test set. Overall, the model achieved high performance across many species, with *Actinomyces israelii*, *Candida albicans*, *Clostridium perfringens*, *Lactobacillus casei*, *Lactobacillus paracasei*, *Listeria monocytogenes*, *Micrococcus* spp., and *Neisseria gonorrhoeae* reaching perfect scores of 1.00 for all three metrics. These results indicate that the network was able to perform error-free classification for species with distinctive morphological features in Gram-stained images.

**Table 2:** Per-class precision, recall, F1-score, and support values for species-level classification on the independent test set.

Bacterium	Precision	Recall	F1-Score	Support
*Acinetobacter baumannii*	1.00	0.80	0.89	5
*Actinomyces israelii*	1.00	1.00	1.00	10
*Bacteroides fragilis*	0.90	0.90	0.90	10
*Bifidobacterium* spp.	0.88	0.88	0.88	8
*Candida albicans*	1.00	1.00	1.00	4
*Clostridium perfringens*	1.00	1.00	1.00	6
*Enterococcus faecalis*	0.88	0.88	0.88	8
*Enterococcus faecium*	1.00	0.86	0.92	7
*Escherichia coli*	0.86	0.86	0.86	7
*Fusobacterium*	0.83	0.62	0.71	8
*Lactobacillus casei*	1.00	1.00	1.00	5
*Lactobacillus crispatus*	1.00	0.20	0.33	5
*Lactobacillus delbrueckii*	0.60	0.60	0.60	5
*Lactobacillus gasseri*	0.88	0.78	0.82	9
*Lactobacillus jensenii*	0.91	0.91	0.91	11
*Lactobacillus johnsonii*	1.00	0.45	0.62	11
*Lactobacillus paracasei*	1.00	1.00	1.00	6
*Lactobacillus plantarum*	0.31	0.80	0.44	5
*Lactobacillus reuteri*	0.83	1.00	0.91	5
*Lactobacillus rhamnosus*	0.50	0.60	0.55	5
*Lactobacillus salivarius*	1.00	0.80	0.89	5
*Listeria monocytogenes*	1.00	1.00	1.00	11
*Micrococcus* spp.	1.00	1.00	1.00	3
*Neisseria gonorrhoeae*	1.00	1.00	1.00	9
*Porphyromonas gingivalis*	0.80	1.00	0.89	8
*Propionibacterium acnes*	0.67	1.00	0.80	4
*Proteus*	0.62	0.83	0.71	6
*Pseudomonas aeruginosa*	1.00	0.33	0.50	3
*Staphylococcus aureus*	0.90	1.00	0.95	9
*Staphylococcus epidermidis*	0.80	0.57	0.67	8
*Staphylococcus saprophyticus*	0.70	0.88	0.78	8
*Streptococcus agalactiae*	1.00	1.00	1.00	4
*Veillonella*	0.75	0.86	0.80	7

Certain species exhibited comparatively lower performance. For example, *Lactobacillus crispatus* and *Pseudomonas aeruginosa* displayed low recall values, suggesting that the model was less effective at correctly identifying positive instances for these taxa. Similarly, *Lactobacillus plantarum* and *Lactobacillus rhamnosus* showed reduced precision and F1-scores, which may be attributed to morphological similarities with closely related species or limited representation in the dataset. Despite these challenges, most taxa achieved F1-scores above 0.80, indicating balanced and reliable classification across the dataset.

Beyond clinical diagnostics, the architectural versatility of our framework suggests high potential for cross-domain applications, including food safety, agricultural monitoring, and environmental microbiology. While the current study primarily validates the model on clinically and industrially relevant taxa, the underlying methodology centered on standard Gram-staining and robust feature extractions are inherently adaptable to microorganisms of diverse origins. In food microbiology, for instance, the rapid identification of spoilage organisms or probiotics could be streamlined using our metadata-enriched reporting. Similarly, in agricultural and environmental settings, the system could be trained to recognize specific microbial indicators of soil health or water contamination. However, transitioning to these settings would require addressing challenges related to complex sample matrices and the potential for greater morphological variability in non-clinical isolates. Future research will focus on expanding the training library with isolates from these ecological niches to evaluate the system’s performance in real-world environmental and agricultural monitoring scenarios.

When aggregated across all classes, the model attained an overall accuracy of 0.84, a weighted precision of 0.88, a weighted recall of 0.84, and a weighted F1-score of 0.84. The Matthews correlation coefficient was also calculated as 0.84, reflecting a high degree of balanced predictive performance. These results confirm the robustness of the CNN in accurately identifying a broad taxonomic range of bacteria, supporting its potential for dependable integration into laboratory diagnostic and research workflows.

**Figure 6:**
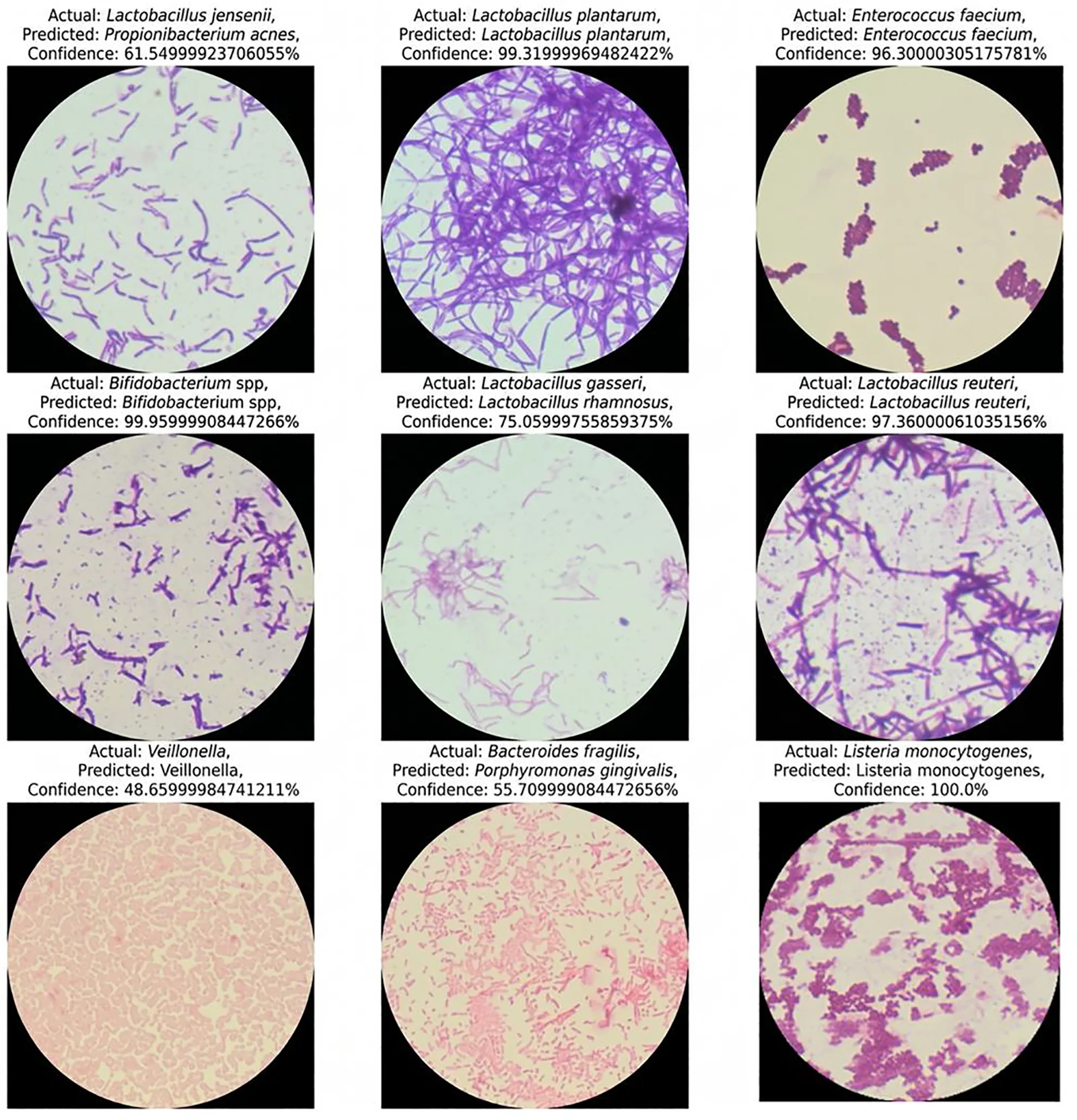
Representative CNN predictions with corresponding bacterial species and confidence scores.

The classification performance of the proposed CNN model was evaluated on a representative set of bacterial microscopy images, as illustrated in **Figure 6**. The model successfully identified several species with high confidence, such as *Lactobacillus plantarum* (99.32%), *Bifidobacterium* spp. (99.96%), *Enterococcus faecium* (96.30%), *Lactobacillus reuteri* (97.36%), and *Listeria monocytogenes* (100.00%). Correct classifications with moderate confidence were also observed, including *Veillonella* (48.66%) and *Lactobacillus gasseri* (75.06%). In certain cases, the model produced misclassifications despite achieving relatively high confidence scores, for example predicting *Propionibacterium acnes* instead of *Lactobacillus jensenii* (61.55%) and *Porphyromonas gingivalis* instead of *Bacteroides fragilis* (55.71%). These findings highlight the model’s strong discriminative ability for most bacterial taxa, while also indicating the presence of challenging instances where interspecies morphological similarities may lead to reduced classification accuracy.

**Figure 7:**
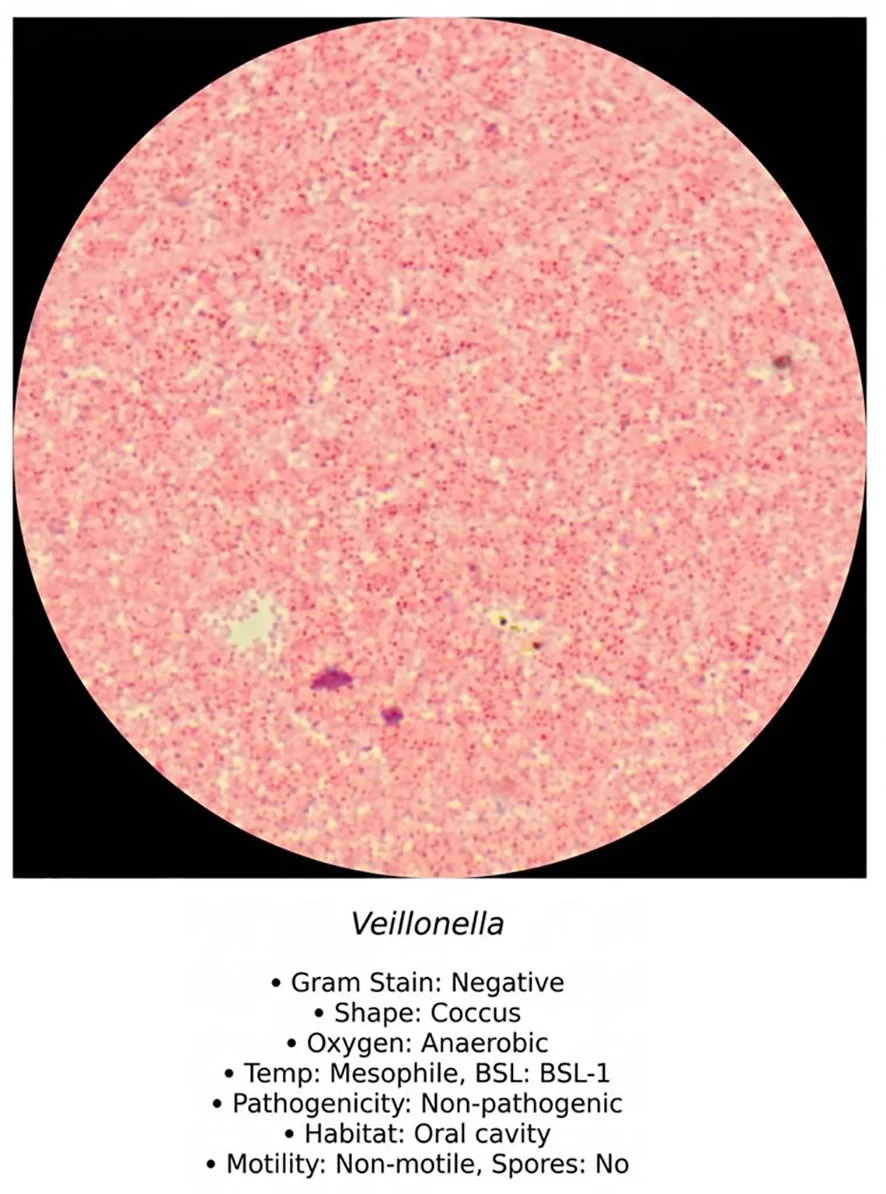
Representative CNN prediction output with integrated phenotypic and ecological metadata for the identified bacterial species.

Upon predicting the species from the microscopy image, the system automatically retrieved the corresponding biological and ecological characteristics from a pre-generated metadata file and displayed them alongside the prediction output. As illustrated in **Figure 7**, the model identified the specimen as *Veillonella*, subsequently presenting its Gram staining properties (negative), cell morphology (coccus), oxygen requirements (anaerobic), optimal growth temperature (mesophile, BSL-1), pathogenicity status (non-pathogenic), typical habitat (oral cavity), and motility features (non-motile, non-spore forming). This combined visual–textual output not only facilitates result interpretation but also supports rapid microbiological assessment by providing essential phenotypic and biosafety information directly linked to the classification outcome.

While the current framework successfully classifies 33 distinct species, the taxonomic representation of fungi was limited to *Candida albicans* in this initial implementation. We acknowledge that expanding the model to include a broader range of yeasts such as the emerging multidrug-resistant pathogen *Candida auris*, or industrially vital species like *Saccharomyces cerevisiae* is a logical next step. Including these species would enhance the system’s clinical utility, particularly in differentiating between morphologically similar non-albicans *Candida* taxa. However, such an expansion necessitates the acquisition of diverse, high-resolution training datasets that reflect the unique blastoconidia and pseudohyphae variations across these species. The modular nature of our metadata-integrated pipeline is designed to facilitate this scaling, allowing for the integration of additional fungal classes as standardized image repositories grow.

## 4 Discussion

The results confirm the technical robustness of the approach, with the CNN achieving strong performance across all species in the dataset. This integration not only enhances the interpretability and practical relevance of predictions but also supports faster, more standardized workflows in microbiological laboratories. The following discussion situates these findings within the context of automated microorganism identification, compares the system’s performance with existing microscopy-based methods, and explores potential extensions for broader clinical and research applications. While the results demonstrate strong predictive performance and practical applicability, several limitations should be acknowledged. Foremost among these is that the proposed framework is strictly designed for and evaluated on single-species cultures (monocultures). The current sequential CNN pipeline lacks the capacity to process polymicrobial or mixed-culture images where multiple distinct bacterial or fungal taxa occupy the same microscopic field of view. In routine clinical diagnostic settings, complex clinical matrices often present mixed infections that require simultaneous multi-taxa identification. Consequently, running the current model on raw mixed-culture micrographs without prior isolation could lead to ambiguous or dominant-class prediction errors.

To overcome this constraint, our future research directions will focus on transitioning the core architecture from a standard image classifier to an object detection and instance segmentation framework (such as YOLO or Mask R-CNN). This evolution will enable the system to dynamically bound, isolate, and individually classify co-existing morphological entities within a single digital specimen. Additionally, addressing these constraints will require the inclusion of larger and more heterogeneous datasets sourced from multiple laboratories, as well as the integration of domain adaptation strategies to enhance robustness. A critical limitation of this architecture is its performance variance across different microbial groups. While the model achieves perfect precision and recall (1.00) for visually distinct taxa like *Candida albicans* and *Actinomyces israelii*, its performance drops significantly within the *Lactobacillus* genus (*L. crispatus* recall of 0.20 and *L. plantarum* precision of 0.31). This drop is not a random error; it stems from a biological limitation of standard Gram-stained 2D microscopy. Under standard light microscopes, different *Lactobacillus* species look almost identical they are all rod-shaped, grow in overlapping clusters, and lack clear geometric differences that a standard CNN model can easily separate at 224 x 224 pixels. Therefore, our findings show that relying only on basic visual features from standard 2D images is not enough for reliable species-level identification within complex genera. To solve these visual ambiguities, future models must either focus on advanced texture analysis or directly integrate non-visual biological properties (like oxygen requirements or growth conditions) into the neural network layers.

These values indicate balanced predictive performance across classes, with the model avoiding significant bias toward overrepresented taxa. When examined on a per-species basis, the CNN achieved perfect scores (precision, recall, F1-score = 1.00) for multiple morphologically distinctive taxa such as *Actinomyces israelii*, *Candida albicans*, *Clostridium perfringens*, *Listeria monocytogenes*, and *Neisseria gonorrhoeae*. These results underscore the model’s capacity to capture discriminative morphological cues inherent to the Gram-staining process, even across phylogenetically distant microorganisms.

The strong agreement between training and validation accuracy curves, coupled with the consistently declining loss trends, indicates effective learning without substantial overfitting. The ROC analysis further confirmed this stability, revealing high AUC values for most species and demonstrating that the classifier maintained high sensitivity and specificity across a wide taxonomic range. In a diagnostic laboratory context, such balanced performance is critical, as both false negatives (risking missed detection of pathogenic bacteria) and false positives (potentially leading to unnecessary interventions) can have significant consequences.

Despite the overall strong performance, some species, including *Lactobacillus crispatus*, *Lactobacillus plantarum*, *Lactobacillus rhamnosus*, and *Pseudomonas aeruginosa*, exhibited reduced precision or recall values. Several factors may account for these discrepancies. First, intra-genus morphological similarities particularly among *Lactobacillus* spp. can hinder visual discrimination when solely relying on Gram-stained features. Second, class imbalance and limited representation of certain taxa in the training dataset may reduce the model’s exposure to within-class variability, thereby affecting generalization. Similar challenges have been reported in previous deep learning–based microbiological classification studies, where morphologically convergent species often require complementary biochemical or genetic data for definitive identification.

The integration of phenotypic and ecological metadata into the prediction output represents a novel and practical extension of the CNN-based framework. By automatically appending attributes such as Gram reaction, cell shape, oxygen requirement, biosafety level, and habitat information to the classification result, the system bridges the gap between automated image analysis and actionable microbiological insight. This feature can significantly enhance interpretability for laboratory personnel, streamline reporting, and facilitate rapid risk assessment particularly in settings where biosafety considerations must be addressed promptly. Comparable metadata-enriched approaches have been proposed in other domains, such as plant disease diagnostics and histopathological image analysis, but have not been widely implemented in bacteriological microscopy classification. From a broader perspective, the model’s ability to handle 33 bacterial taxa with relatively high accuracy positions it as a promising candidate for deployment in clinical microbiology laboratories, research facilities, and educational environments. It is important to note that while metadata integration significantly enhances the interpretability of results, it acts primarily as a post-classification decision-support tool rather than a direct correction mechanism for the CNN’s visual errors. To further mitigate misclassifications among morphologically convergent taxa, such as *Lactobacillus* species, future iterations of this framework could employ a hierarchical classification strategy. In this approach, a specialized sub-network could be triggered specifically for the most challenging genera to capture subtle intra-genus textural features. Furthermore, moving beyond a sequential reporting system toward a ‘multimodal fusion’ model where phenotypic parameters like oxygen requirement and growth temperature are fed directly into the fully connected layers alongside visual features. This would allow the system to reach a diagnostic conclusion by fusing visual representations and phenotypic data points in a single computational step.

A study demonstrated that MobileNet-based CNN models outperformed corneal specialists in differentiating bacterial and fungal corneal ulcers from images alone (AUC 0.83–0.86), showing potential for earlier diagnosis and targeted treatment (Redd et al., 2022). A study introduced a microscopy-based framework combining 3D quantitative phase imaging and neural networks to identify 19 bacterial species from single or few cells with up to 99.9% accuracy, offering a faster alternative to traditional microbial identification for early infection treatment (Kim et al., 2022). Another study proposed a digital twinning approach for bacteria using deep transfer learning models such as MobileNetV2, EfficientNetV2-S, and ResNet-50, achieving up to 99.33% test accuracy and enabling accurate, rapid, and virtual bacterial identification for clinical and metaverse applications (Jamshidi et al., 2023b). Moreover, a study introduced an automated deep learning-based bacterial classification method using polyculture microscopy images and a ResNet50 architecture, achieving 94.91% accuracy and demonstrating its potential to reduce diagnostic time and costs in clinical microbiology (Sarker et al., 2024).

When compared with previous studies, the present framework demonstrates a balanced combination of accuracy, generalizability, and practical integration features. Several prior works in this domain have achieved higher numerical accuracies for a smaller set of bacterial classes or under highly controlled imaging conditions. For example, the study utilizing 3D quantitative phase imaging and neural networks reported classification accuracies approaching 99.9 percent; however, its scope was limited to nineteen bacterial species and relied on specialized imaging equipment that may not be readily available in standard diagnostic laboratories. Similarly, the digital twinning approach employing advanced deep transfer learning models attained test accuracies above 99 percent, yet the dataset characteristics and imaging protocols were optimized for maximum separation between classes, potentially reducing applicability in real-world microscopy workflows where staining variability and morphological overlap are common.

In contrast, the present system operates on standard Gram-stained microscopy images acquired under diverse conditions from multiple sources, encompassing thirty-three bacterial taxa with a broad range of morphological and physiological traits. While the overall accuracy of 0.84 is lower than the peak values reported in some highly optimized studies, the performance was achieved in a setting that better reflects operational variability in microbiological laboratories. Furthermore, unlike most prior works, this framework incorporates a metadata enrichment component that delivers immediate contextual information about Gram reaction, morphology, biosafety level, habitat, and other laboratory-relevant traits. This integration addresses a practical gap by linking visual recognition directly to actionable microbiological insight, thereby supporting rapid risk assessment and decision-making.

Compared with studies that focused solely on binary or limited multi-class differentiation, such as the MobileNet-based system for distinguishing bacterial from fungal corneal ulcers, the present work tackles a more complex taxonomic landscape while maintaining consistent predictive balance across classes. This breadth of coverage, combined with the emphasis on interpretability and operational compatibility, positions the system as a robust candidate for deployment in clinical and research environments where both accuracy and contextual relevance are essential.

To provide a clearer and more comprehensive assessment of the proposed integrated diagnostic framework, a systematic benchmarking against recently developed state-of-the-art networks is provided in **Table 3**. This structural comparison highlights the technical and operational trade-offs between absolute numerical accuracy, hardware dependency, taxonomic coverage, and workflow interpretability.

**Table 3:** Comparative analysis of the proposed framework against previously developed deep learning approaches for microbial identification.

Study	Input/modality	Taxonomic scope	Advantages	Disadvantages/limitations	Hardware & computational footprint	Target deployment environment
Redd et al. (2022) (MobileNet)	Clinical Keratitis Images	Binary (Bacterial vs. Fungal)	High diagnostic relevance for targeted ulcer treatment; outperformed specialists.	• Limited binary resolution; cannot provide species-level bacterial tracking.	Lightweight network; optimized for mobile clinical screens.	Specialized point-of-care ophthalmic clinics.
Kim et al. (2022) (Neural Network)	3D Quantitative Phase Imaging (QPI)	19 Bacterial Species	• Near-perfect accuracy (up to 99.9%) from a minute quantity of cells. • Rapid execution.	• High reliance on complex, non-standard, and expensive imaging hardware not commonly found in clinical labs.	Highly intensive phase-reconstruction algorithms; requires specialized processing units.	Advanced research institutes and specialized central facilities.
Jamshidi et al. (2023b) (MobileNetV2, EfficientNetV2-S, ResNet-50)	Digital Twin/Microorganism Images	33 Taxa	• Excellent test accuracy (99.33%). • Tailored for virtual/metaverse applications.	• Dataset was optimized for maximum separation, potentially oversimplifying real-world staining and hardware variations.	Heavy deep transfer learning architectures; high VRAM/GPU footprint during training.	High-performance workstation environments/Virtual labs.
Sarker et al. (2024) (ResNet50)	Polyculture Microscopy Images	Species-Level	• Reduces diagnostic costs and time in polyculture environments. • High accuracy (94.91%).	• Does not provide post-prediction contextual support or ecological reporting for downstream lab execution.	Standard ResNet-50 backbone; requires intermediate local GPU acceleration.	Digitally modernized, well-funded clinical diagnostic labs.
Proposed Framework (Sequential CNN + Metadata Repository)	Standard Gram-Stained Microscopy Images	33 Taxa (including 32 bacteria & 1 yeast)	• Low hardware barrier: Operates on standard, accessible microscopy images. • Context-Aware: Uniquely bridges image analysis and lab workflows by auto-generating 8-feature metadata reports. • High robustness against operational/staining variability.	• Lower peak accuracy (84.0%) compared to networks trained under highly controlled, pristine imaging environments. • Lower performance on morphologically convergent intra-genus species (*Lactobacillus* spp.).	Ultra-lightweight framework; optimized to operate with zero GPU acceleration/low memory footprints.	Resource-limited settings, standard field laboratories, and local educational centers.

To contextualize the technical value of the proposed sequential CNN architecture, a baseline benchmarking against established deep learning backbones utilized in recent microbiological vision pipelines was executed as detailed in **Table 3**. While contemporary state-of-the-art architectures such as ResNet-50, EfficientNetV2-S, or heavy phase-reconstruction networks achieve superior top-1 accuracies under pristine, heavily regularized experimental setups, they inherently possess substantially higher parameter counts, massive training footprints, and strict hardware dependencies. Our custom sequential CNN was intentionally designed as a lightweight architecture to demonstrate that robust species-level classification (0.84 accuracy) can be maintained without advanced, high-cost computing components. As structured in **Table 3**, this architectural simplification ensures that the operational and economic deployment barriers remain low, enabling the pipeline to operate efficiently on standard, non-GPU-accelerated desktop computing infrastructure typically found in resource-constrained regional laboratories, while seamlessly delivering integrated metadata decision support.

While the results demonstrate strong predictive performance and practical applicability, several limitations should be acknowledged. The dataset, although taxonomically diverse, does not fully represent the range of morphological variability that can occur in clinical or environmental isolates. Rare or atypical morphotypes, as well as species with closely overlapping Gram-stained features, may present classification challenges that reduce generalizability. In addition, the model was trained and validated using images captured under standardized laboratory conditions, which may not fully reflect the variability in staining quality, illumination, and imaging hardware encountered across different institutions. Addressing these constraints in future work will require the inclusion of larger and more heterogeneous datasets sourced from multiple laboratories, as well as the integration of domain adaptation strategies to enhance robustness.

We acknowledge that the current framework operates as a sequential pipeline, where metadata retrieval functions as a post-classification decision-support tool rather than a direct mathematical priors layer within the neural network optimization process. While this sequential integration significantly streamlines the laboratory reporting workflow by bridging visual inference with clinical context, it does not constitute a multimodal integrated learning model. To achieve a true computational study, future iterations of this framework will investigate multimodal fusion architectures. In such advanced networks, non-visual clinical metadata (such as oxygen requirements, optimal growth temperatures, and anatomical isolation sites) will be tokenized and injected directly into the dense layers of the network alongside the flattened visual feature maps. This approach will allow the optimization loss function to be constrained simultaneously by both morphological and physiological data points, thereby creating a tightly coupled hybrid learning framework capable of resolving highly convergent intra-genus visual ambiguities.

Future developments may focus on combining the current visual classification framework with complementary molecular or spectroscopic data, enabling a truly multimodal diagnostic approach that could improve accuracy for morphologically similar taxa. In particular, integrating 16S rRNA gene sequencing data directly into the proposed framework as a parallel confirmation layer represents a highly promising avenue. By cross-referencing the CNN’s image-based morphological predictions and curated metadata with precise genomic sequences, the system can transition into a comprehensive, high-fidelity diagnostic pipeline. Such an integrated tool would extend beyond routine clinical triage, serving as a robust decision-support platform that directly assists the precise analytical workflows of both microbiologists and molecular biologists. Furthermore, real-time deployment studies in clinical microbiology workflows will be essential to evaluate operational efficiency, user acceptance, and the overall impact on diagnostic decision-making.

## 5 Conclusion

This study demonstrates the feasibility and effectiveness of a convolutional neural network–based framework for species-level bacterial classification from Gram-stained microscopy images, enhanced with metadata-driven contextual reporting. By integrating high-resolution image analysis with curated microbiological attributes, the system not only achieves robust classification performance across diverse taxa but also delivers interpretable outputs that align with laboratory needs. The results highlight the model’s potential for accelerating microbial identification, standardizing reporting, and supporting decision-making in clinical, research, and educational contexts. While certain species with close morphological similarities remain challenging to distinguish, the framework’s modular architecture allows for future integration of larger datasets, advanced deep learning models, and complementary data modalities such as genomic sequencing or spectroscopic profiling. Overall, this work represents a step toward fully automated, multimodal microbiological diagnostic systems capable of improving efficiency, accuracy, and accessibility in both high-resource and resource-limited settings.

## Data Availability

The raw data supporting the conclusions of this article will be made available by the authors, without undue reservation.

## References

[B1] Abd S. M. Soliman N. F. Eldin S. Abd Elrahman S. E. Ismail N. A. El-Rabaie E. S. M. . (2020). Bacterial classification with convolutional neural networks based on different data reduction layers. Nucleosides Nucleotides Nucleic Acids 39, 493–503. doi: 10.1080/15257770.2019.1645851 31418627

[B2] Adekoya A. Okezue M. A. Menon K. (2025). Medical laboratories in healthcare delivery: A systematic review of their roles and impact. Laboratories 2, 8. doi: 10.3390/laboratories2010008 30654563

[B3] Ahmad S. Lohiya S. Taksande A. Meshram R. J. Varma A. Vagha K. . (2024). A comprehensive review of innovative paradigms in microbial detection and antimicrobial resistance: beyond traditional cultural methods. Cureus 16. doi: 10.7759/cureus.61476 38952583 PMC11216122

[B4] Ali M. Benfante V. Basirinia G. Alongi P. Sperandeo A. Quattrocchi A. . (2025). Applications of artificial intelligence, deep learning, and machine learning to support the analysis of microscopic images of cells and tissues. J. Imaging 11, 59. doi: 10.3390/jimaging11020059 39997561 PMC11856378

[B5] Altheide S. T. (2020). Biochemical and culture-based approaches to identification in the diagnostic microbiology laboratory. Am. Soc. For. Clin. Lab. Sci. 32(4), 166–175. doi: 10.29074/ascls.2019001875

[B6] Chaturvedi N. Yadav M. K. Sharma M. (2024). “ Applications of artificial intelligence and machine learning in microbial diagnostics and identification,” in Methods in Microbiology, vol. 55. ( Elsevier), 213–230.

[B7] Ching T. Himmelstein D. S. Beaulieu-Jones B. K. Kalinin A. A. Do B. T. Way G. P. . (2018). Opportunities and obstacles for deep learning in biology and medicine. J. R. Soc Interface 15, 20170387. doi: 10.1098/rsif.2017.0387 29618526 PMC5938574

[B8] Dargan S. Kumar M. Ayyagari M. R. Kumar G. (2020). A survey of deep learning and its applications: a new paradigm to machine learning. Arch. Comput. Methods Eng 27, 1071–1092. doi: 10.1007/s11831-019-09344-w 30311153

[B9] De Medici D. Kuchta T. Knutsson R. Angelov A. Auricchio B. Barbanera M. . (2015). Rapid methods for quality assurance of foods: the next decade with polymerase chain reaction (PCR)-based food monitoring. Food Anal. Methods 8, 255–271. doi: 10.1007/s12161-014-9915-6 30311153

[B10] Ferone M. Gowen A. Fanning S. Scannell A. G. M. (2020). Microbial detection and identification methods: Bench top assays to omics approaches. Compr. Rev. Food Sci. Food Saf 19, 3106–3129. doi: 10.1111/1541-4337.12618 33337061

[B11] Galperin M. Y. (2018). What bacteria want. Environ. Microbiol. 20, 4221–4229. doi: 10.1111/1462-2920.14398 30187651 PMC7020242

[B12] Gow N. A. R. Johnson C. Berman J. Coste A. T. Cuomo C. A. Perlin D. S. . (2022). The importance of antimicrobial resistance in medical mycology. Nat. Commun. 13, 5352. doi: 10.1111/1462-2920.14398 36097014 PMC9466305

[B13] Gradisteanu Pircalabioru G. Raileanu M. Dionisie M. V. Lixandru-Petre I.-O. Iliescu C. (2024). Fast detection of bacterial gut pathogens on miniaturized devices: an overview. Expert Rev. Mol. Diagn 24, 201–218. doi: 10.1080/14737159.2024.2316756 38347807

[B14] Hassall J. Coxon C. Patel V. C. Goldenberg S. D. Sergaki C. (2024). Limitations of current techniques in clinical antimicrobial resistance diagnosis: examples and future prospects. NPJ Antimicrobials Resist 2, 16. doi: 10.1038/s44259-024-00033-8 39843577 PMC11721362

[B15] Huang Y. Sheth R. U. Zhao S. Cohen L. A. Dabaghi K. Moody T. . (2023). High-throughput microbial culturomics using automation and machine learning. Nat. Biotechnol. 41, 1424–1433. doi: 10.1038/s41587-023-01674-2 36805559 PMC10567565

[B16] Huo D. Wang X. (2024). A new era in healthcare: The integration of artificial intelligence and microbial. Med. Novel Technol. Devices 23, 100319. doi: 10.1016/j.medntd.2024.100319 38826717

[B17] Ishaq S. L. Parada F. J. Wolf P. G. Bonilla C. Y. Carney M. A. Benezra A. . (2021). Introducing the microbes and social equity working group: considering the microbial components of social, environmental, and health justice. MSystems 6, 10–1128. doi: 10.1128/msystems.00471-21 34313460 PMC8407420

[B18] Ishaq Z. Faiz F. Haider U. Ali A . (2025). “ Artificial intelligence and machine learning techniques applied to microbiology,” in Microbiology in the Era of Artificial Intelligence ( CRC Press), 271–290. doi: 10.1201/9781003410164-15

[B19] Jamshidi M. (. Sargolzaee S. Foorginezhad S. Moztarzadeh O. (2023a). Bacteria data for machine vision and digital biology. Mendeley Data V1. doi: 10.17632/cvkgfzp7ck.1

[B20] Jamshidi M. (. Sargolzaei S. Foorginezhad S. Moztarzadeh O. (2023b). Metaverse and microorganism digital twins: a deep transfer learning approach. Appl. Soft Comput. 147, 110798. doi: 10.1016/j.asoc.2023.110798 38826717

[B21] Jeckel H. Drescher K. (2021). Advances and opportunities in image analysis of bacterial cells and communities. FEMS Microbiol. Rev. 45, fuaa062. doi: 10.1093/femsre/fuaa062 33242074 PMC8371272

[B22] Jiang Y. Luo J. Huang D. Liu Y. Li D. D. (2022). Machine learning advances in microbiology: a review of methods and applications. Front. Microbiol. 13. doi: 10.3389/fmicb.2022.925454 35711777 PMC9196628

[B23] Kim G. Ahn D. Kang M. Park J. Ryu D. Jo Y. . (2022). Rapid species identification of pathogenic bacteria from a minute quantity exploiting three-dimensional quantitative phase imaging and artificial neural network. Light Sci. Appl. 11, 190. doi: 10.1038/s41377-022-00881-x 35739098 PMC9226356

[B24] Kotwal S. Rani P. Arif T. Manhas J. Sharma S. (2022). Automated bacterial classifications using machine learning based computational techniques: architectures, challenges and open research issues. Arch. Comput. Methods Eng 29, 2469–2490. doi: 10.1007/s11831-021-09660-0 34658617 PMC8505783

[B25] Kreiss L. Jiang S. Li X. Xu S. Zhou K. C. Lee K. C. . (2023). Digital staining in optical microscopy using deep learning-a review. PhotoniX 4, 34. doi: 10.1186/s43074-023-00113-4 38164791

[B26] Lawrence J. R. Korber D. R. Neu T. R. (2007). “ Analytical imaging and microscopy techniques,” in Manual of Environmental Microbiology, 40–68.. doi: 10.1128/9781555815882.ch5

[B27] Lowe S. E. Jain M. K. Zeikus J. G. (1993). Biology, ecology, and biotechnological applications of anaerobic bacteria adapted to environmental stresses in temperature, pH, salinity, or substrates. Microbiol Rev. 57, 451–509. doi: 10.1128/mr.57.2.451-509.1993 8336675 PMC372919

[B28] Mumuni A. Mumuni F. (2021). CNN architectures for geometric transformation-invariant feature representation in computer vision: a review. SN Comput. Sci. 2, 340. doi: 10.1007/s42979-021-00735-0 30311153

[B29] Rani P. Kotwal S. Manhas J. Sharma V. Sharma S. (2022). Machine learning and deep learning based computational approaches in automatic microorganisms image recognition: methodologies, challenges, and developments. Arch. Comput. Methods Eng 29, 1801–1837. doi: 10.1007/s11831-021-09639-x 34483651 PMC8405717

[B30] Rawson T. M. Wilson R. C. O’Hare D. Herrero P. Kambugu A. Lamorde M. . (2021). Optimizing antimicrobial use: challenges, advances and opportunities. Nat. Rev. Microbiol. 19, 747–758. doi: 10.1038/s41579-021-00578-9 34158654

[B31] Redd T. K. Prajna N. V. Srinivasan M. Lalitha P. Krishnan T. Rajaraman R. . (2022). Image-based differentiation of bacterial and fungal keratitis using deep convolutional neural networks. Ophthalmol. Sci. 2, 100119. doi: 10.1016/j.xops.2022.100119 36249698 PMC9560557

[B32] Sarker I. A. Khan M. R. R. Prova S. I. Khan M. I. Morshed M. A. Reza A. W. . (2024). “ Utilizing deep learning for microscopic image based bacteria species identification”, in: 2024 IEEE International Conference on Computing, Applications and Systems (COMPAS), 1–5. doi: 10.1109/COMPAS60761.2024.10796040

[B33] Shamir L. Delaney J. D. Orlov N. Eckley D. M. Goldberg I. G. (2010). Pattern recognition software and techniques for biological image analysis. PloS Comput. Biol. 6, e1000974. doi: 10.1371/journal.pcbi.1000974 21124870 PMC2991255

[B34] Traore B. B. Kamsu-Foguem B. Tangara F. (2018). Deep convolution neural network for image recognition. Ecol. Inf. 48, 257–268. doi: 10.1016/j.ecoinf.2018.10.002 38826717

[B35] Treebupachatsakul T. Poomrittigul S. (2019). “ Bacteria classification using image processing and deep learning”, in: 2019 34th International Technical Conference on Circuits/Systems, Computers and Communications (ITC-CSCC), Computers and Communications (ITC-CSCC), JeJu, Korea (South) 1–3. doi: 10.1109/ITC-CSCC.2019.8793320

[B36] Yang Z. Tian Y. Zhang J. (2024). Adaptive image processing embedding to make the ecological tasks of deep learning more robust on camera traps images. Ecol. Inf. 82, 102705. doi: 10.1016/j.ecoinf.2024.102705 38826717

[B37] Zhang Y. Jiang H. Ye T. Juhas M. (2021). Deep learning for imaging and detection of microorganisms. Trends Microbiol. 29, 569–572. doi: 10.1016/j.tim.2021.01.006 33531192

[B38] Zieliński B. Plichta A. Misztal K. Spurek P. Brzychczy-Włoch M. Ochońska D. (2017). Deep learning approach to bacterial colony classification. PloS One 12, e0184554. doi: 10.1371/journal.pone.0184554 28910352 PMC5599001

